# Supplementary material for: Universal model of individual and population mobility on diverse spatial scales
Source: Nat Commun. 2017 Nov 21;8:1639. doi: 10.1038/s41467-017-01892-8 (PMC5696346; doi:10.1038/s41467-017-01892-8)
Supplement: Supplementary file 1 — Supplementary Information [file 41467_2017_1892_MOESM1_ESM.pdf]

SUPPLEMENTARY INFORMATION

Supplementary Figures

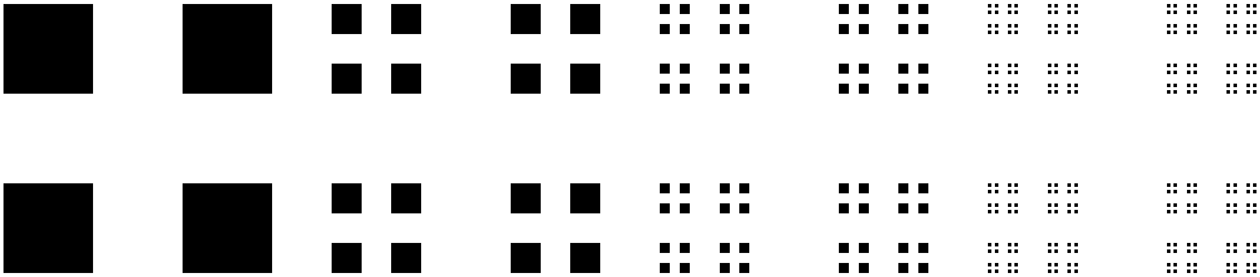

Supplementary Figure 1 | The first 4 iterations in the generation of a 2D Cantor dust.

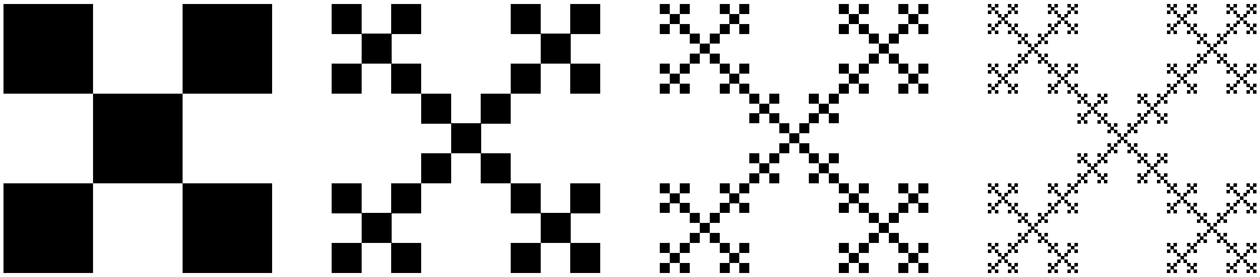

Supplementary Figure 2 | The first 4 iterations of the Vicsek fractal.

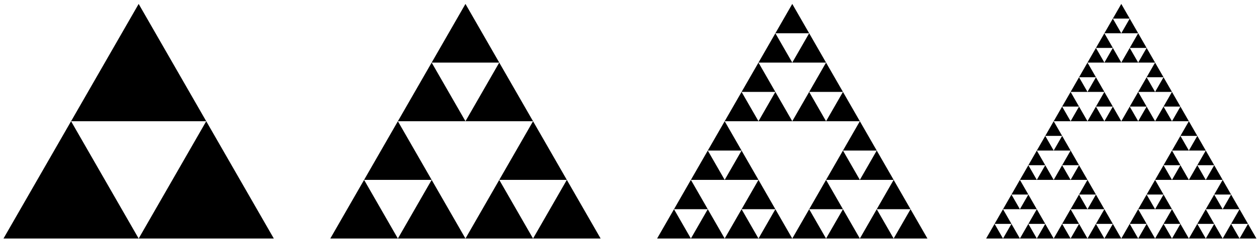

Supplementary Figure 3 | The first 4 iterations of the Sierpinski triangle.

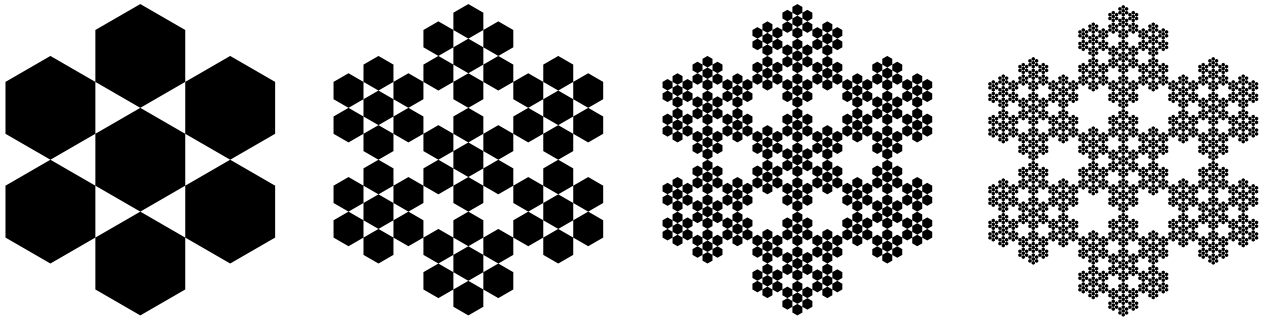

Supplementary Figure 4 | The first 4 iterations of the hexaflake.

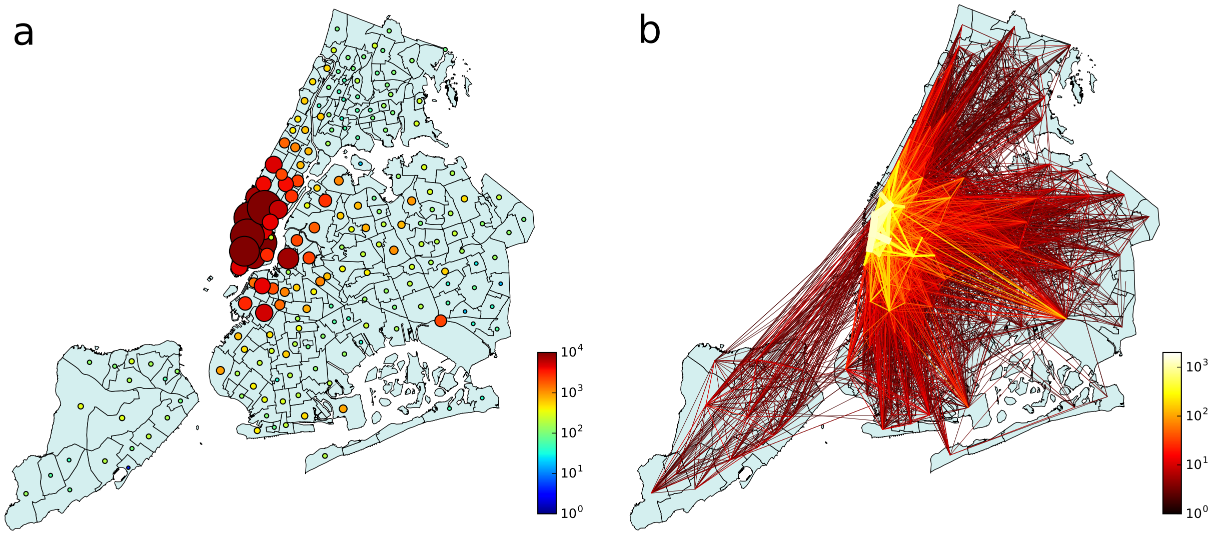

Supplementary Figure 5 | Foursquare users check-ins and mobility data in New York city. (a) Check-in times distribution. The zones are U.S. 2010 census blocks. (b) Traveling steps between locations.

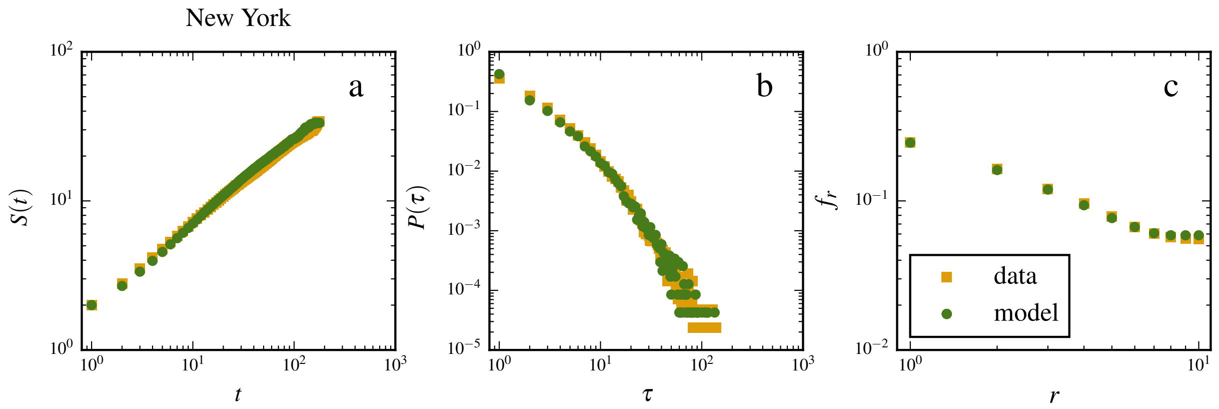

Supplementary Figure 6 | Model predicted and empirical statistical patterns of individual mobility in New York city. (a) The total number $S(t)$ of locations visited as an increasing function of time t, (b) the return time distribution $P(\tau)$, and (c) Zipf-like frequency distribution of location visits. In all panels, the green color specifies results predicted from our model, and orange denotes the empirical results obtained directly from data.

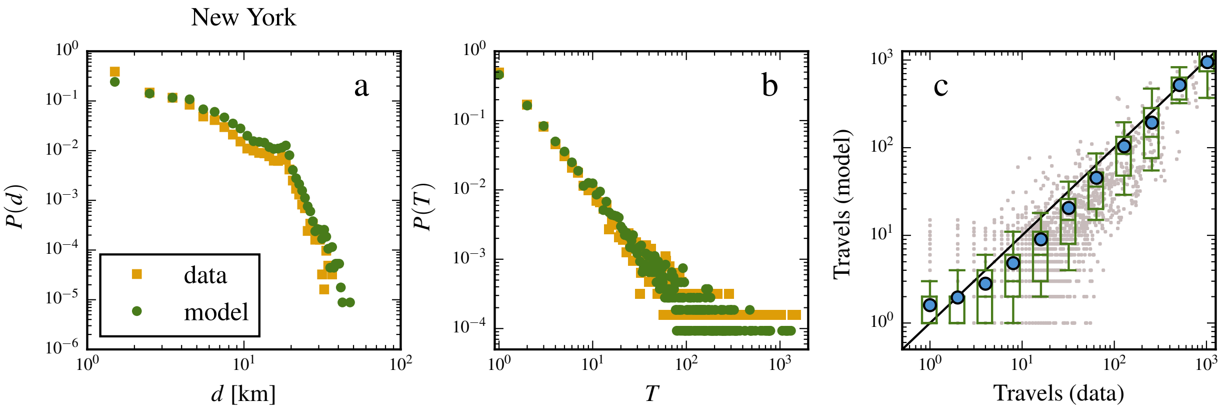

Supplementary Figure 7 | Model predicted and empirical statistical patterns of collective mobility in New York city. Model predicted (green circles) and real (orange squares) distributions of (a) traveling distance d and (b) the number of traveling steps, T, between two locations. (c) Model predicted versus real values of T. The grey points are scatter plot for each pair of locations. The blue points represent the average number of predicted travels in different bins. The standard boxplots represent the distribution of the number of predicted travels in different bins of the number of observed travels. A box is marked in green if the line $y = x$ lies between 10% and 91% in that bin and in red otherwise.

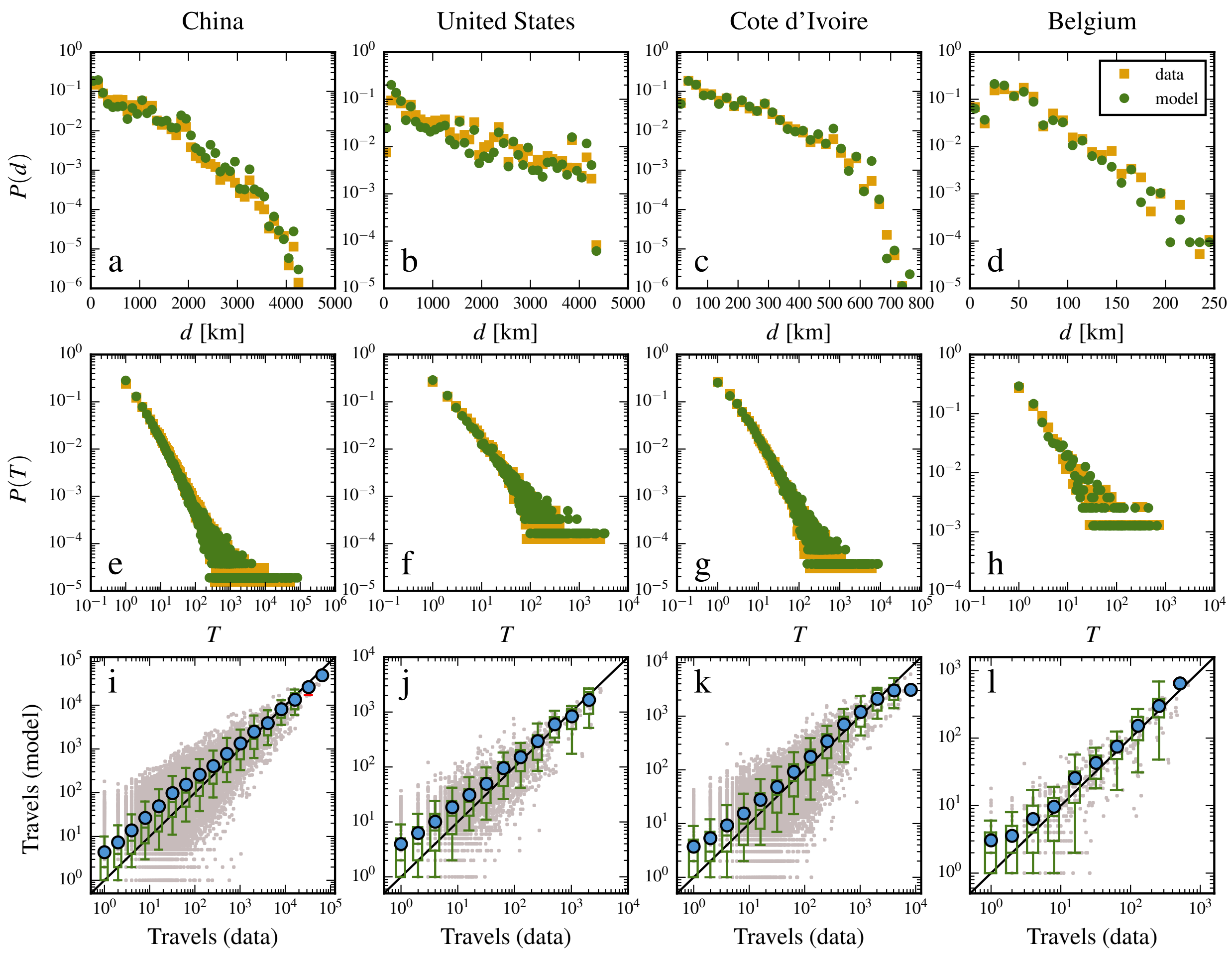

Supplementary Figure 8 | Collective mobility patterns predicted by the memory-free model. The variables and symbols represent the same meanings as that in Supplementary Fig. 7.

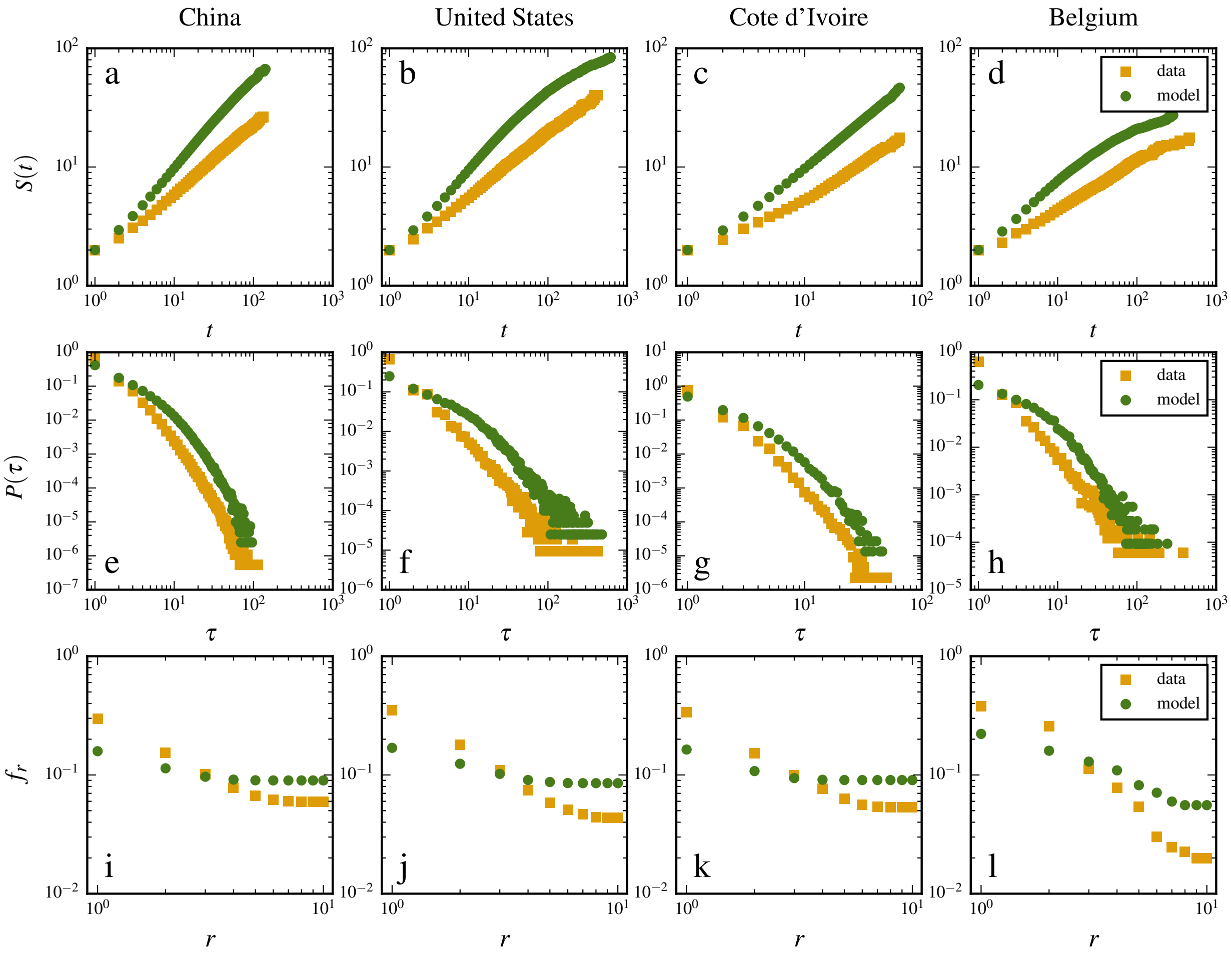

Supplementary Figure 9 | Individual mobility patterns predicted by the memory-free model.

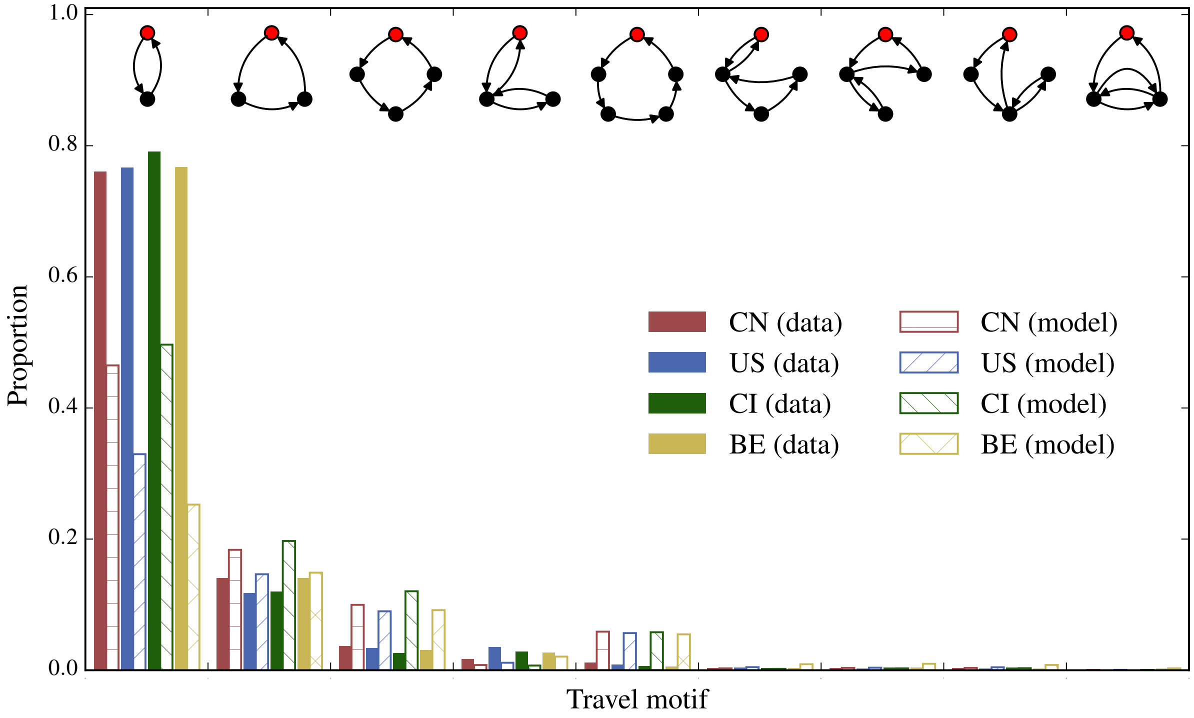

Supplementary Figure 10 | Frequency of occurrence of motifs in the memory-free model.

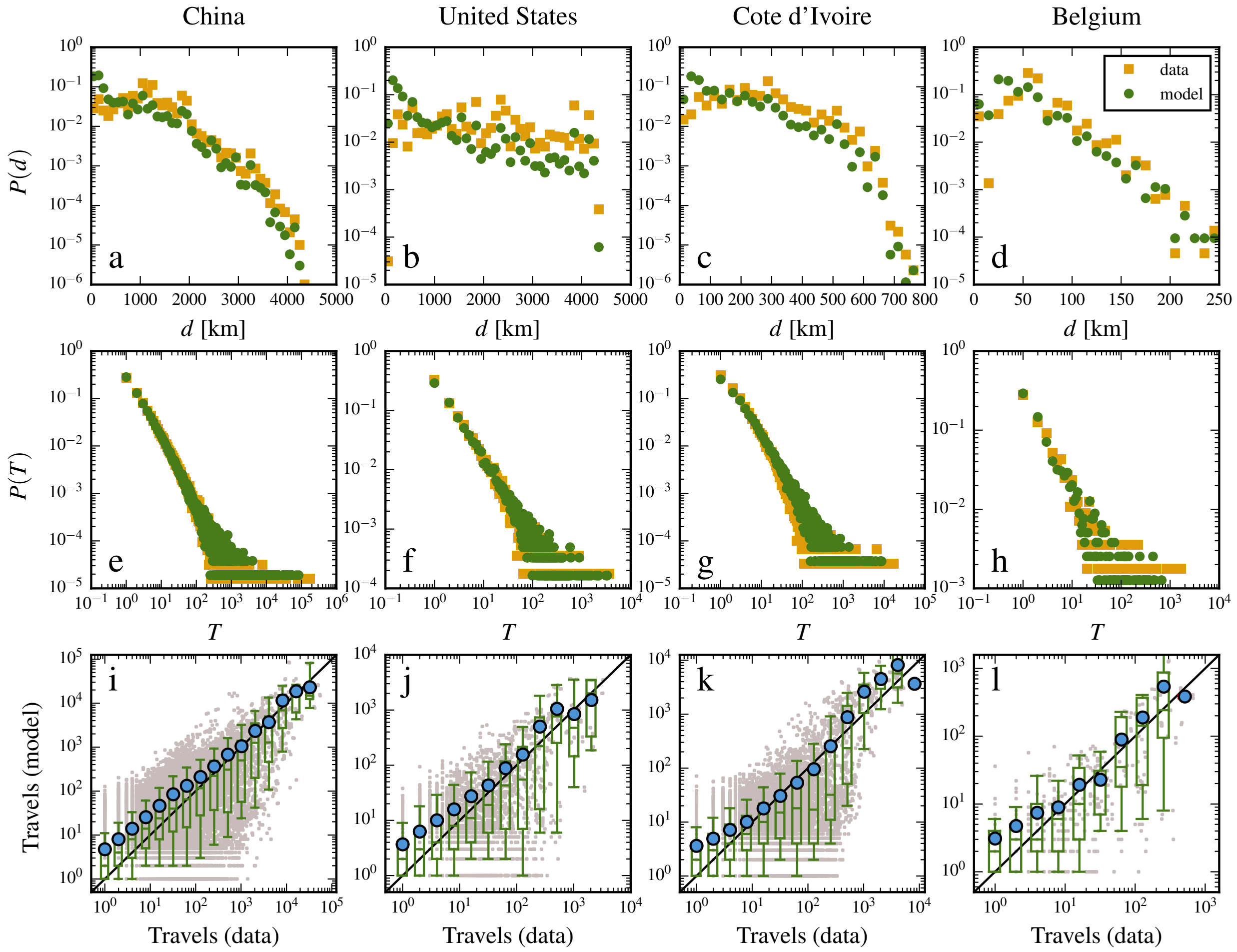

Supplementary Figure 11 | Collective mobility patterns predicted by the competition-free model. The variables and symbols represent the same meanings as that in Supplementary Fig. 7.

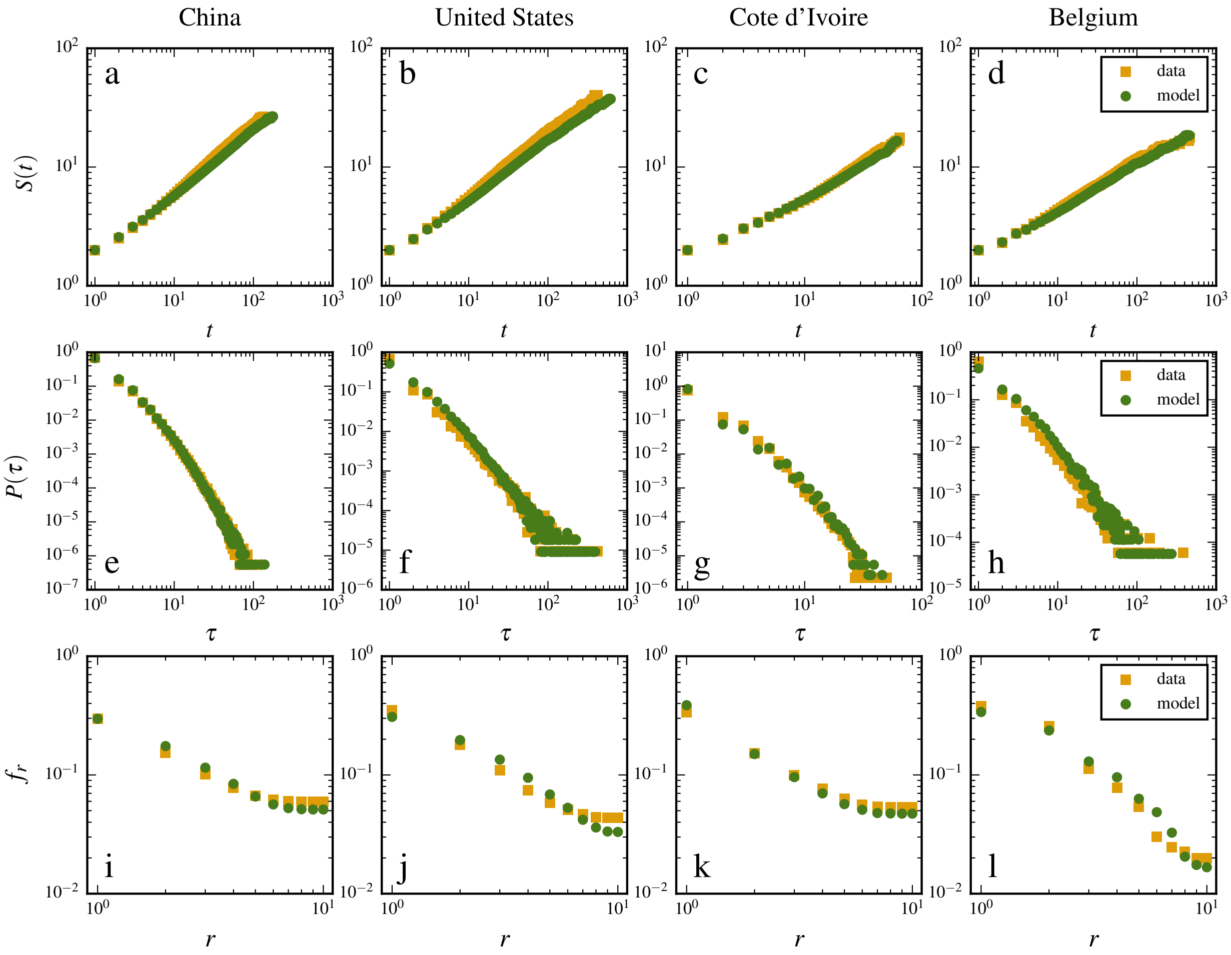

Supplementary Figure 12 | Individual mobility patterns predicted by the competition-free model.

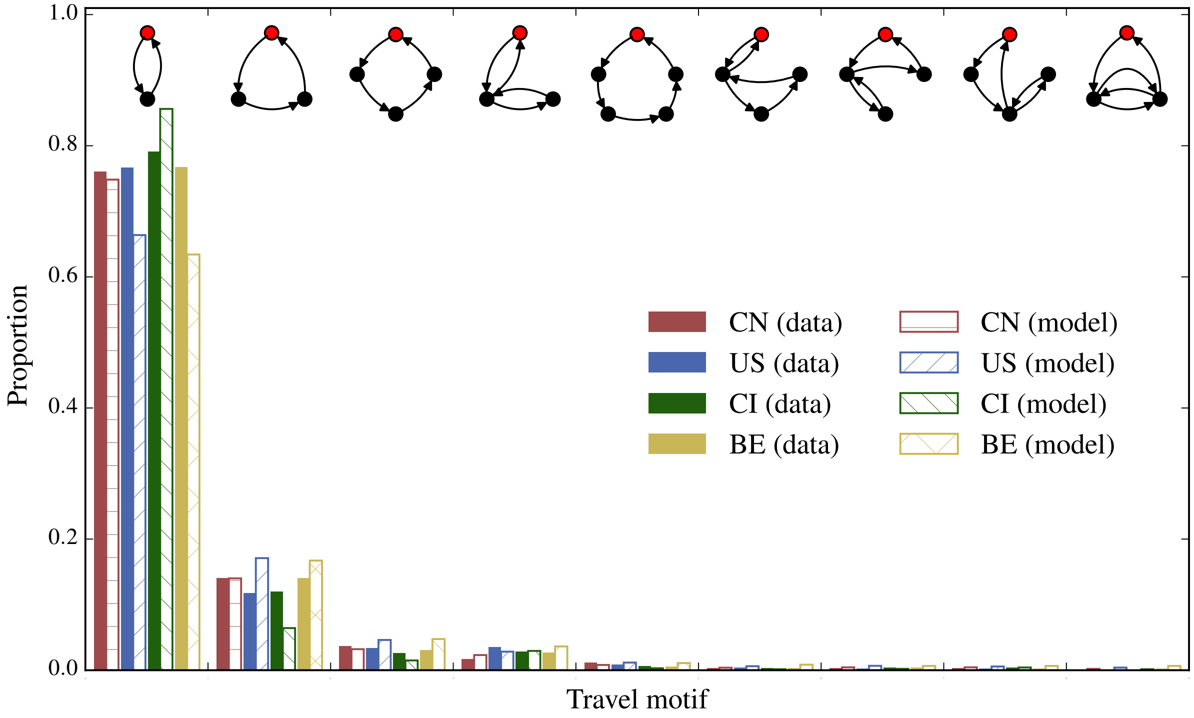

Supplementary Figure 13 | Frequency of occurrence of motifs in the competition-free model.

Supplementary Table

Supplementary Table 1 | Comparison of models prediction accuracy. SSI is the Sørensen similarity index between real observations of collective travel flow and model results. RMSE is the root-mean-square error of predicted $S(t)$. OM, MF, and CF stand for the original model, the memory-free model, and the competition-free model, respectively.

Country	SSI-OM	SSI-MF	SSI-CF	RMSE-OM	RMSE-MF	RMSE-CF
China	0.7126	0.6759	0.5394	1.3556	24.8674	1.6098
US	0.7013	0.7225	0.5156	1.8376	29.3604	4.7381
Cote d'Ivoire	0.7136	0.6914	0.5333	0.6738	16.7012	0.6926
Belgium	0.6936	0.7739	0.5481	0.7141	9.2657	0.8308

Supplementary Notes

Supplementary Note 1: Analysis of individual mobility patterns

1.1 Individual mobility model based on Zipf's law

We consider a simplified version of our human mobility model, which is obtained by imposing the approximations that the population is uniformly distributed among all available locations and the effects of population induced competition are negligible. The simplified model is effectively a random walk with memory in a finite space, in which the transition probability p_{ij} of an individual traveling form location i to j is

$$p_{ij} \propto 1 + \frac{\lambda}{r_j}, \tag{1}$$

where λ is the memory strength parameter and r_j denotes that j is the rth newly visited location for the individual. Insofar as λ is much larger than unity, the transition probability approaches that given by the basic Zipf's law: $p_{ij} \propto \lambda^{-1} + r_j^{-1} \approx r_j^{-1}$.

We first consider the function $S(t)$, the number of locations that an individual has visited in time t. The probability of choosing a new city at the next time step is

$$P_{\text{new}} = \frac{N - S}{N + \sum_{r=1}^{S} \lambda N / r}, \tag{2}$$

where N is the total number of cities and r signifies that the corresponding location is the rth newly visited city. Treating S as continuous, for $S \ll N$, we have

$$P_{\text{new}} \approx \frac{1}{1 + \lambda \int_1^S \frac{\mathrm{d}r}{r}} = \frac{1}{1 + \lambda(\ln S + C)}, \tag{3}$$

where C is the Euler's constant and $C \approx 0.577$. We thus get

$$\frac{\mathrm{d}S}{\mathrm{d}t} = P_{\text{new}} = \frac{1}{1 + \lambda(\ln S + C)}, \tag{4}$$

which gives

$$t = (1 + \lambda C)S + \lambda S(\ln S - 1) - A. \tag{5}$$

Since $S(t=0)=1$, we obtain $A = 1+\lambda C - \lambda$. Substituting this relation into Eq. (5) yields

$$t = AS + \lambda S \ln S - A, \tag{6}$$

which determines the function $S(t)$ implicitly. For $\lambda = 0$ (i.e., no memory effect), we have $S = t+1$. For $\lambda = 1/(2-C)$, we have $\lambda = A$, so Eq. (6) can be written as $t = \lambda[S + S\ln(S) - 1]$. Using the approximation $\ln(S) \approx S-1$, we get

$$S \sim \sqrt{(2-C)t+1}, \tag{7}$$

indicating that S increases sublinearly with t.

We next derive the frequency distribution f_r of visited locations. Since $S(t)$ distinct locations have been visited by time t, the probability for an individual to choose an already visited location is

$$P_{\text{old}} = 1 - P_{\text{new}} = \frac{S + \sum_{r=1}^{S} \lambda N/r}{N + \sum_{r=1}^{S} \lambda N/r}. \tag{8}$$

Among all the $S(t)$ old locations, the probability of visiting the rth location, i.e., the rate of increase of g_r, the number of times that the location has been visited, is

$$\frac{\mathrm{d}g_r}{\mathrm{d}t} = P_{\text{old}} \frac{1+\lambda N/r}{S + \sum_{r=1}^{S} \lambda N/r} = \frac{1+\lambda N/r}{N + \sum_{r=1}^{S} \lambda N/r} \approx \frac{\lambda}{r[1+\lambda(\ln S + C)]}. \tag{9}$$

From Eq. (6), we have $\mathrm{d}t = [A + \lambda(\ln S + 1)]\mathrm{d}S$, which when being substituted into Eq. (9) yields

$$\frac{\mathrm{d}g_r}{\mathrm{d}S} = \frac{\lambda}{r}. \tag{10}$$

We thus obtain

$$g_r = \lambda S/r + B. \tag{11}$$

For $S=1$, we have $r=1$ and $g_r = 1$, so the constant B in Eq. (11) is given by $B = 1-\lambda$. Since f_r is proportional to g_r, we have

$$f_r \propto \lambda S/r + B. \tag{12}$$

We finally analyze the return time distribution, $P(\tau)$, the probability that a traveler returns to a previously visited location after τ steps. In our model, for the rth first visited location, $P_r(\tau)$ is the joint probability of the following three probabilities: (1) q_r, the probability of visiting the location at the present, (2) $(1-q_r)^{\tau-1}$, the probability that the rth location will

not be visited in the remaining $\tau-1$ time steps, and (3) q_r, the probability that the location is visited exactly at time τ. We have

$$P_r(\tau) = q_r^2(1-q_r)^{\tau-1}. \quad (13)$$

From Eq. (9), the probability that the rth location is visited at each time step is given by

$$q_r = \frac{H}{r}, \quad (14)$$

where $H = \lambda/[1+\lambda(\ln S + C)]$. Substituting Eq. (14) into Eq. (13), we get

$$P_r(\tau) = \frac{H^2}{r^2}\left(1-\frac{H}{r}\right)^{\tau-1}. \quad (15)$$

For all the already visited locations, the return time distribution is given by

$$P(\tau) = \int_1^S P_r(\tau)\mathrm{d}r = \int_1^S \frac{H^2}{r^2}\left(1-\frac{H}{r}\right)^{\tau-1}\mathrm{d}r = \frac{H}{\tau}\left[\left(1-\frac{H}{S}\right)^{\tau} - (1-H)^{\tau}\right], \quad (16)$$

which is approximately a mixture of two algebraic terms of the identical exponent -1.

1.2 Individual mobility model based on the generalized Zipf's law

The above simplified model assumes that individuals visit locations with the probability given by the basic Zipf's law. However, there are situations where individuals choose their visiting locations by following the generalized Zipf's law [1]: $f \propto 1/r^{\zeta}$ ($\zeta > 1$). We are thus led to incorporate the generalized Zipf's law into our model to gain a better understanding of the real mobility behavior at the individual level. A straightforward way to generalize our basic model is to modify the transition probability from $p_{ij} \propto 1+\lambda/r_j$ to $p_{ij} \propto 1+\lambda/r_j^{\zeta}$, where ζ is an adjustable parameter. To be able to derive analytical results on the individual mobility patterns, we further simplify the transition probability to be $p_{ij} \propto 1/r^{\zeta}$. In addition, to obtain theoretical results associated with the generalized Zipf's law, it is necessary to exploit the exploration and preferential return (EPR) model proposed by Song et al. [2]. In the EPR model, an individual has two choices when he/she chooses a location: (1) exploration - the individual moves to a new location with the probability $P_{\mathrm{new}} = \rho S^{-\gamma}$, where S is the total number of visited locations with ρ and γ being parameters; (2) preferential return - the individual returns to a previously visited location with the probability $1-P_{\mathrm{new}}$, and the

probability to visit an old location is proportional to the number of travels to this location. The EPR model can lead analytically to the generalized Zipf's law from the relation $P_{\mathrm{new}} = \rho S^{-\gamma}$. As an inverse process of the EPR model, the basic assumption of our model is the generalized Zipf's law, and we aim at analytically deriving the relationship between P_{new} and S. Our generalized model can be constructed, as follows.

There are two options for an individual at each step: (1) travel to a new location with the probability P_{new} that decreases with the increase of S and approaches 0 for large values of S; (2) returns to an old location with the probability $1 - P_{\mathrm{new}}$, and the probability of selecting the i-th old location is proportional to the number g_i of travels to location i. We assume that the visiting frequency f_r to the rth visited location follows the generalized Zipf's law

$$f_r \propto r^{-\zeta}, \tag{17}$$

where $\zeta > 1$ is an adjustable parameter. The frequency f_i of visiting the location i can be calculated by the number of visits to all the locations:

$$f_i = \frac{g_i}{t}, \tag{18}$$

where t is the total number of travel steps (time steps), which is equal to the total number of visits to all locations $t = \sum_{i=1}^{S} g_i$. When a location i was first visited, we have

$$g_i(t_i) = 1, \tag{19}$$

where t_i is the time at which location i was discovered. For a large value of S, the probability P_{new} approaches 0, so the rate of increase in the number of visits to the i-th location is approximately given by

$$\frac{\mathrm{d}g_i}{\mathrm{d}t} = \frac{g_i}{t}, \tag{20}$$

for which the solution is

$$g_i = C_i t, \tag{21}$$

where C_i is an arbitrary constant. Combining Eqs. (19) and (21), we have

$$g_i(t_i) = C_i t_i = 1, \tag{22}$$

and

$$C_i = \frac{1}{t_i}. \tag{23}$$

Inserting Eq. (23) into Eq. (21), we obtain

$$g_i = \frac{t}{t_i}. \tag{24}$$

Combining Eqs. (17), (18) and (24), we get

$$t_r \propto r^{\zeta}, \tag{25}$$

where t_r denotes the travel time during which location r was first visited.

For a trajectory with t jumps and S distinct locations, we can rank all visited locations $r = 1, 2, ..., S$ in the order of the discovery time t_r. We then have

$$S(t_r) = r. \tag{26}$$

Combining Eqs. (25) and (26), we obtain

$$S(t_r) \propto t_r^{\frac{1}{\zeta}}, \tag{27}$$

or

$$S(t) \propto t^{\frac{1}{\zeta}}, \tag{28}$$

which gives the scaling relation between time t and the visited location number $S(t)$. Note that $S(t) \propto t^{\frac{1}{\zeta}}$ is the solution of

$$\frac{\mathrm{d}S}{\mathrm{d}t} \propto S^{1-\zeta}. \tag{29}$$

Since the increase rate of S equals the probability of visiting the new location, i.e.,

$$P_{\mathrm{new}} = \frac{\mathrm{d}S}{\mathrm{d}t}, \tag{30}$$

we obtain

$$P_{\mathrm{new}} = \rho S^{-(\zeta-1)}, \tag{31}$$

where ρ is a parameter and can be determined from empirical data [2]. These results demonstrate that, according to the assumption that the visiting frequency follows the generalized Zipf's law, our generalized model can give rise to a power-law relationship between P_{new} and S. If we denote $\zeta = 1 + \gamma$, Eq. (31) becomes

$$P_{\mathrm{new}} = \rho S^{-\gamma}, \tag{32}$$

which is exactly the same as the basic assumption - the relationship between P_{new} and S in the EPR model [2]. This analytical result indicates that the generalized Zipf's law and the

power-law relation between P_{new} and S have a mutually causal relationship, so the model for individual mobility based on the former is equivalent to that based on the latter.

We also derive the return time distribution $P(\tau)$. For a sufficiently large value of S, the probability that the rth location is visited at each time step is

$$q_r \propto f_r \propto \frac{1}{r^{\zeta}} \tag{33}$$

or

$$q_r = \frac{L}{r^{\zeta}}, \tag{34}$$

where L is a constant and $\sum_1^S L/r^{\zeta} = 1$. Combining Eqs. (13), (15) and (34), we can formulate the probability that an individual returns to the rth visited location after τ steps as

$$P_r(\tau) = \frac{L^2}{r^{2\zeta}}\left(1 - \frac{L}{r^{\zeta}}\right)^{\tau-1}. \tag{35}$$

For $\zeta \approx 1$, we have $P_r(\tau) \approx \frac{L^2}{r^2}(1 - \frac{L}{r})^{\tau-1}$, so

$$P(\tau) = \int_1^S P_r(\tau)\mathrm{d}r \approx \frac{L}{\tau}\left[\left(1 - \frac{L}{S}\right)^{\tau} - (1 - L)^{\tau}\right]. \tag{36}$$

For $\zeta \gg 1$, $P(\tau)$ cannot be solved analytically but numerical solutions can be readily obtained.

Supplementary Note 2: Analysis of collective mobility patterns

2.1 Uniform population distribution

To derive the mobility patterns at the population level, we simplify the model further by assuming that each individual move only one step. In this case, the individual memory effect can be neglected, so the transition probability p_{ij} of population traveling form location i to j is

$$p_{ij} \propto \frac{m_j}{W_{ji}}. \tag{37}$$

If there are m individuals at each location and all locations are uniformly distributed in a 2-D domain, we have $W_{ji} = \rho\pi d_{ij}^2$, where ρ is the population density. In addition, using $T_{ij} = m_i p_{ij}$, we can rewrite Eq. (37) as

$$T_{ij} \propto \frac{m_i m_j}{d_{ij}^2}, \tag{38}$$

which is the gravity model with a square distance function. In the real world, the spatial distribution of cities in a region often exhibits fractal properties [3]: $W_{ji} \propto d_{ij}^D$, where D is the fractal dimension. We thus have

$$T_{ij} \propto \frac{m_i m_j}{d_{ij}^D}, \tag{39}$$

which is the gravity model with an algebraic distance function.

Since the populations at different locations are assumed to be equal, Eq. (39) can be simplified as

$$T(d) \propto d^{-D}, \tag{40}$$

which is the number T of traveling steps between two locations of distance d apart. We then obtain the travel distance distribution as

$$P(d) \propto d^{-D}, \tag{41}$$

which is an algebraic distribution with exponent $-D$. In the real world the fractal dimension D ranges from 1 to 2 [3]. Indeed a previous work [4] demonstrated that the empirical scaling exponents from some real travel distance distributions are consistent with the value of the fractal dimension.

From Eq. (40), we obtain the distance between two locations as a function of T as

$$d(T) \propto T^{-\frac{1}{D}}. \tag{42}$$

Moreover, the number of location pairs $N(d)$ with distance $\leq d$ in a fractal space is

$$N(d) \propto Q(y \leq d) \propto d^D, \tag{43}$$

where $Q(y \leq d)$ is the fraction of location pairs with distance $\leq d$ in the fractal space. The quantity $Q(y \leq d)$ is larger than or equal to the fraction of location pairs with traveling steps $T(d)$, for the reason that the distance d is a decreasing function of T, as indicated by Eq. (40). We thus have

$$P(x \geq T) = Q[y \leq d(T)]. \tag{44}$$

Combining Eqs. (42), (43) and (44), we obtain the distribution of T as

$$P(T) \propto \frac{\mathrm{d}d(T)^D}{\mathrm{d}T} \propto \frac{\mathrm{d}(T^{-\frac{D}{D}})}{\mathrm{d}T} \propto T^{-2}, \tag{45}$$

which is an algebraic distribution with exponent -2 - a universal value independent of the fractal dimension D of the domain.

2.2 Heterogeneous population distribution

In the real world, population distributions among cities can be highly heterogeneous, which has a significant effect on the distribution of T. To treat the heterogeneity analytically, we consider a simple scenario in which the travelers are only allowed to travel from the central city c to other cities. The central city has the largest population m_c, and the population m_j of another city decreases from m_c algebraically as a function of the distance d_{cj} to the central city:

$$m_j \propto d_{cj}^{-\xi}, \tag{46}$$

with $\xi > 0$. Combining Eqs. (37) and (46), we obtain

$$T_{cj} \propto \frac{m_c m_j}{W_{jc}} \propto \frac{m_c d_{cij}^{-\xi}}{\bar{m} d_{cj}^{D}} \propto d_{cj}^{-D-\xi}, \tag{47}$$

where $\bar{m}$ is the average population of the cities. Analogously, Eqs. (43), (44) and (47) lead to

$$P(T) \propto \frac{\mathrm{d}(T^{-\frac{D}{D+\xi}})}{\mathrm{d}T} \propto T^{-1-\frac{D}{D+\xi}}, \tag{48}$$

where the scaling exponent ranges from -1 to -2, covering most empirically observed exponent values in the real-world travel flow distributions [see Fig. 5(e-h) in the main text].

Supplementary Note 3: Simulation setup of mobility behaviors in a fractal domain

To validate our analytical results, we simulate our simplified model in four typical fractal domains: 2D Cantor dust, Vicsek fractal, Sierpinski triangle, and hexaflake. The results are

shown in Fig. 6(d) in the main text. Here we describe the four fractal sets and the simulation setup.

3.1 2D Cantor dust

The Cantor dust [5] is a 2D version of the Cantor set. It is obtained by starting with a basic unit square, scaling its size by 1/3, then placing four scaled copies at the four corners, respectively. Recursively applying the procedure *ad infinitum*, we generate a 2D Cantor set, as shown in Supplementary Fig. 1. Let N_n be the number of black boxes and L_n be the length of a side of a box after the nth iteration. The fractal dimension of the Cantor set is

$$D = -\lim_{n\to\infty} \frac{\ln N_n}{\ln L_n} = -\frac{\ln 4^n}{\ln 3^{-n}} = \frac{\ln 4}{\ln 3} \approx 1.262. \tag{49}$$

3.2 Vicsek fractal

The Vicsek fractal [6], also known as the box fractal, can be constructed using a procedure similar to that for the 2D Cantor dust. In particular, the basic unit square is decomposed into nine smaller squares in a 3-by-3 grid. The four squares at the corners and the middle square are kept, while the other squares are removed. The process is repeated recursively for each of the five remaining subsquares. The Vicsek fractal is the set obtained at the limit of this procedure, as shown in Supplementary Fig. 2. Its fractal dimension is

$$D = -\lim_{n\to\infty} \frac{\ln N_n}{\ln L_n} = -\frac{\ln 5^n}{\ln 3^{-n}} = \frac{\ln 5}{\ln 3} \approx 1.465. \tag{50}$$

3.3 Sierpinski triangle

The Sierpinski triangle [7], or Sierpinski gasket, is obtained by starting from an equilateral triangle of unit side length (in fact any triangle can be used), subdividing it into four smaller congruent equilateral triangles, and removing the central one. This procedure is repeated *ad infinitum*, generating the Sierpinski triangle fractal, as shown in Supplementary Fig. 3. Its fractal dimension is

$$D = -\lim_{n\to\infty} \frac{\ln N_n}{\ln L_n} = -\frac{\ln 3^n}{\ln 2^{-n}} = \frac{\ln 3}{\ln 2} \approx 1.585. \tag{51}$$

3.4 Hexaflake

Hexaflake [8] is a fractal constructed starting from a hexagon, which is replaced by a flake of smaller hexagons such that a scaled hexagon is placed at each vertex and at the center. This procedure is repeated recursively to generate the hexaflake, as shown in Supplementary Fig. 4, with its fractal dimension given by

$$D = -\lim_{n\to\infty} \frac{\ln N_n}{\ln L_n} = -\frac{\ln 7^n}{\ln 3^{-n}} = \frac{\ln 7}{\ln 3} \approx 1.771. \tag{52}$$

3.5 Simulation setup

We iterate the construction procedure for each of the four fractal geometries and set the central position of the shapes in each fractal set (black blocks in Supplementary Fig. 1-4) to be the location coordinates. To avoid an idealized fractal domain and better mimic empirically observed fractal features in the real world, we add a small random offset to the coordinates of each location. The iterations are 5, 4, 6, 3 for the 2D Cantor dust, Vicsek fractal, Sierpinski triangle and hexaflake, respectively, and the numbers of existent shapes (locations) are 1024, 625, 729 and 343, respectively.

We set population m at each location and use Eq. (37) to calculate the number of traveling steps T_{ij} between any location pairs. We use $\min(T)$ to rescale all T, i.e., we let the minimum number of traveling steps be one, and finally obtain the travel flow distribution $P(T)$. Similarly, we can obtain the rescaled travel distance distribution $P(d)$, as shown in Fig. 6(d) in the main text.

Supplementary Note 4: Model validation with empirical mobility data at the city scale

In the main text we have demonstrated that our model is universally applicable to countries with diverse spatial scales. Here we show that the model can also characterize individual and population mobility patterns at the city scale accurately.

We use the foursquare check-ins data set [9] in New York city as the proxy data of human mobility for model validation. The data set contains 42035 individuals, in which 23520 have travels among different locations (here the locations are defined as the 2010 census blocks [10], see Supplementary Fig. 5), and the total number of traveling steps is 113279.

For this data set, we first estimate the memory strength parameter and obtain $\lambda \approx 8.0$. We then simulate our model using this parameter value and compare the results with those from the real mobility data, as shown in Supplementary Figs. 6 and 7. We see that both the individual and collective mobility patterns in New York city can be predicted, suggesting that our model is capable of characterizing human mobility patterns at small (intra-city) spatial scales.

Supplementary Note 5: Comparison with alternative models

Our mobility model contains two essential ingredients: individual memory and population induced competition effects. They jointly determine the attractiveness of a location, as well as the transition probability for an individual to move from one location to another. Here we consider two alternative models, a memory-free model and a competition-free model, to show that the two ingredients are indispensable for modeling human mobility patterns.

5.1 Memory-free model

To construct a memory-free model, we modify Eq. (3) in the main text by writing the transition probability p_{ij} of traveling from location i to j as

$$p_{ij} \propto \frac{m_j}{W_{ji}}. \tag{53}$$

The model prediction results are shown in Supplementary Figs. 8, 9 and 10. We see that, while the memory-free model can reproduce the collective mobility patterns to certain extent, it fails to capture the individual movement patterns.

To offer a better comparison of the results obtained from different models, we use two indices to measure the model prediction accuracy. The first is the Sørensen similarity index (SSI) [11], which is a statistical tool to identify the similarity between two samples. Here we use SSI to quantify the degree of similarity between real observations of collective travel flow between locations and model prediction results. The SSI is defined as

$$\mathrm{SSI} \equiv \frac{1}{N^2}\sum_{i}^{N}\sum_{j}^{N}\frac{2\min(T'_{ij}, T_{ij})}{T'_{ij}+T_{ij}}, \tag{54}$$

where T'_{ij} is the model predicted traveling steps from location i to j and T_{ij} is the observed number. Apparently, if each T'_{ij} is equal to T_{ij} the index should be unity, whereas if all T'_{ij}s are far from the real values, the index should be close to 0.

The second index is the root-mean-square error (RMSE) [12], which is a frequently used quantity to measure the differences between the model predicted and actually observed values. We use RMSE to measure the prediction errors of the models in the total number of locations $S(t)$ visited within time t, which is an important characteristic of individual movement patterns. The RMSE of $S(t)$ is defined as

$$\mathrm{RMSE} = \sqrt{\frac{\sum_{t=1}^{L_{\max}}[S_{\mathrm{real}}(t) - S(t)]^2}{L_{\max}}}, \tag{55}$$

where $S_{\mathrm{real}}(t)$ is obtained from the actual data set, $S(t)$ is calculated from the model, and $L_{\max}$ is the maximum time step.

We calculate the SSI between observed and predicted travel flow and RMSE of the predicted $S(t)$ for the original model and memory-free model, and list the results in Supplementary Table 1. These results suggest that the memory effect mainly affects the individual movement patterns but has a little effect on the collective mobility patterns.

5.2 Competition-free model

The competition-free model is a memory-based mobility model without any population induced competition effect. In this model, the transition probability p_{ij} of traveling from location i to j is

$$p_{ij} \propto m_j(1+\frac{\lambda}{r_j}), \tag{56}$$

meaning that individuals only consider the inherent attractiveness of locations and their own preferences induced by memory when selecting travel destinations.

Supplementary Figs. 11, 12, 13 and Supplementary Table 1 show the prediction results from the competition-free model. We see that, in contrast to the memory-free model, the competition-free model can reasonably reproduce the individual mobility patterns, but the accuracy of predicting collective movement patterns is poor. *These results further demonstrate that both the memory effect and population induced competition effect are essential for modeling and predicting human mobility patterns simultaneously at the individual and population levels.*

Supplementary References

[1] R. E. Wyllys, Empirical and theoretical bases of Zipf's law. *Library Trends* **30**, 53-64 (1981).

[2] C. Song, T. Koren, P. Wang, A. L. Barabási, Modelling the scaling properties of human mobility. *Nat. Phys.* **6**, 818-823 (2010).

[3] P. Frankhauser, The fractal approach. A new tool for the spatial analysis of urban agglomerations. *Population* **10**, 205-240 (1998).

[4] R. Gallotti, A. Bazzani, S. Rambaldi, M. Barthelemy, A stochastic model of randomly accelerated walkers for human mobility. *Nat. Commun.* **7**, 12600 (2016).

[5] G. Helmberg, *Getting Acquainted with Fractals* (Walter de Gruyter, 2007).

[6] T. Vicsek, M. Shlesinger, M. Matsushita, *Fractals in Natural Sciences* (World Scientific, 1994).

[7] A. A. Lale, B. V. Khiste, G. Burshe, S. Khobragade, Study of Sierpinski triangle gasket. In 2011 3rd International Conference on Electronics Computer Technology (ICECT), Vol. 3, pp. 386-390 (2011).

[8] S. M. Choudhury, M. A. Matin, Effect of FSS ground plane on second iteration of hexaflake fractal patch antenna, In 2012 7th International Conference on Electrical Computer Engineering (ICECE), pp. 694-697 (2012).

[9] J. Bao, Y. Zheng, F. M. Mokbel, Location-based and Preference-Aware Recommendation Using Sparse Geo-Social Networking Data. In ACM SIGSPATIAL (GIS 2012), Redondo Beach, CA, US, 2012

[10] Data can be accessed via http://gis.nyc.gov/census/

[11] T. Sørensen, A method of establishing groups of equal amplitude in plant sociology based on similarity of species and its application to analyses of the vegetation on danish commons. *Biol. Skr.* **5** 1-34 (1948).

[12] R. J. Hyndman, A. B. Koehler, Another look at measures of forecast accuracy. *Int. J. Forecast.* **22**, 679-688 (2006).
