# Supplementary material for: Universal model of individual and population mobility on diverse spatial scales
Source: Nat Commun. 2017 Nov 21;8:1639. doi: 10.1038/s41467-017-01892-8 (PMC5696346; doi:10.1038/s41467-017-01892-8)
Supplement: Supplementary file 3 — Description of Additional Supplementary Files [file 41467_2017_1892_MOESM3_ESM.pdf]

Description of Additional Supplementary Files

File Name: Supplementary Data 1
Description: Longitude and latitude coordinates of all prefecture-level cities in mainland China.

File Name: Supplementary Data 2
Description: Calculated individual mobility trajectories in mainland China using user check-in records of social networks in China.
